# Malaria testing and treatment knowledge among selected rural patent and proprietary medicine vendors (PPMV) in Nigeria

**DOI:** 10.1186/s12936-019-2732-z

**Published:** 2019-03-27

**Authors:** Oladimeji Oladepo, Abisoye S. Oyeyemi, Musibau A. Titiloye, Adedayo O. Adeyemi, Sarah M. Burnett, Iorwakwagh Apera, Opeyemi Oladunni, Michael Alliu

**Affiliations:** 1 Department of Health Promotion and Education, Faculty of Public Health, College of Medicine, University of Ibadan, Ibadan, Nigeria; 2 Department of Community Medicine, Niger Delta University, Wilberforce Island, Bayelsa State Nigeria; 3 Africare, Washington, DC USA; 4 West African Infectious Diseases Institute, Abuja, Nigeria; 5 Adeleke University, Ede, Osun State Nigeria

**Keywords:** Malaria treatment, Patent and proprietary medicine vendors, Diagnosis, Rapid diagnostic tests

## Abstract

**Background:**

Malaria is a leading cause of illness and death in Nigeria, but access of poor people to quality anti-malarial services remains low especially in the rural areas. Patent and proprietary medicine vendors (PPMVs) provide the majority of malaria treatment in rural areas, but little is known about their knowledge of malaria testing and treatment of uncomplicated malaria as recommended in the 2011 National Malaria Control Programme policy.

**Methods:**

A cross-sectional survey was conducted in two purposively selected states (Oyo and Bayelsa) in Nigeria with each state representing a different geographic and linguistic–ethnic region in the southern part of the country. Two rural LGAs were randomly selected from each state and data were collected from 160 randomly selected PPMVS (40 per LGA) using a structured questionnaire. Data were analysed using descriptive statistics.

**Results:**

The 2011 National Policy on Malaria Diagnosis and Treatment is mostly unknown to PPMVs. Although most PPMVs (89%) knew that artemisinin-based combination therapy (ACT) is recommended in the national policy, 91% also thought non-ACT were endorsed. The proportion of PPMVs who stated they would treat a malaria case with an artemisinin-based combination at the correct dose was 33% for a child under five, 47% for an adult male and 14% for a pregnant woman in her second trimester. The proportion of PPMVs who reported they would diagnose a case of malaria prior to treatment using a malaria rapid diagnostic test (RDT) kit was 1.9% for children under five, 7.5% for adult males and 3.1% for pregnant women in their first trimester due to lack of knowledge. Almost two-thirds (65.6%) would correctly refer children with severe malaria to health facility.

**Conclusions:**

Substantial knowledge gaps on the use of RDTs and treatment with artemisinin-based combinations exist among rural PPMVs. Given existing evidence regarding the effectiveness of private retail outlets in malaria case management, PPMVs should be provided with competency-based training and supervision to improve the quality of care they provide.

## Background

Malaria is a leading cause of death among low-income countries [1] and remains a major public health problem worldwide. Malaria and its complications are controlled by preventing infection and prompt diagnosis and effective treatment [2]. Africa accounts for 91% of all malaria deaths and, with an estimated 57.5 million cases and 225,000 deaths per year. Nigeria accounts for 27% of the total African malaria burden [3]. Within Nigeria, malaria is a major cause of illness, death, and poverty, and a significant drain on the economy and wellbeing of the nation. It is estimated that 50% of Nigeria’s adult population will have at least one episode of malaria each year and children under five will have 2–4 attacks annually [4].

Children under five and pregnant women are most at risk for malaria-related morbidity and mortality, with 11% of maternal and 20% of under-five deaths attributed to malaria [5–7]. Poorer and more rural populations are also at greater risk, with malaria prevalence higher among the lowest wealth quintile (42.9% vs. 4.4% among highest quintile) and among rural populations (35.6% vs. 11.5% among urban populations) [5]. In respect to treatment, the proportion of children with fever who were treated with any artemisinin-based combination increased from 12% in 2010 to 18% in 2013 and 38% in 2015 [7, 8].

The private sector in Nigeria provides 60% of the health care for the country and distributes 90% of all anti-malarials [9, 10]. Patent and proprietary medicine vendors (PPMVs), defined broadly as “persons without formal pharmacy training selling orthodox pharmaceutical products on a retail basis for profit” [11] form a significant proportion of the private sector. When faced with symptoms of malaria, 46% of Nigerians choose to go first to chemists and PPMVs [8], hence the need to involve the private sector in malaria drug delivery system in Nigeria.

In Nigeria, under the 2011 National Policy on Malaria Diagnosis and Treatment, PPMVs are allowed to sell anti-malarials [4], and the 2011 NMCP implementation guide for parasite-based diagnosis of malaria recommended that PPMVs provide rapid diagnostic testing (RDT) to clients with fever, prior to prescribing ACT as first-line treatment of uncomplicated malaria [12]. By explicitly including PPMVs in these guidelines, the Federal Government recognized this sector’s contribution to malaria treatment.

Recent studies have shown that most PPMVs have low knowledge of malaria and do not provide appropriate malaria treatment. A 2007 study revealed that 43% of PPMVs knew that there were new malaria treatment guidelines released in 2005 and only 25% could accurately identify the changes in these guidelines [13]. A 2012 study found that 49% could identify government recommended ACT as the best treatment for malaria [14]. Although the quality of services provided by PPMVs is poor, there is some evidence that PPMV knowledge and practice can be improved through interventions [15].

An in-depth study of patent medicine sellers’ perspectives on malaria in a rural Nigerian community identified issues regarding the poor knowledge and poor dispensing behaviour of PPMVs in relation to childhood malaria episodes. The study show that most PPMVs were not trained health professionals and often did not provide appropriate advice and adequate doses of anti-malarial to their clients [16].

The purpose of this study is to describe the current malaria testing and treatment knowledge of rural registered PPMVs in selected states of Nigeria. It is expected that the findings of this study would inform interventions that could improve PPMV’s knowledge in diagnosing and treating malaria. The outcome of the study would add to literature on the contribution of the private sector to the treatment of malaria.

## Methods

### Study design

This is a cross-sectional survey conducted in two purposively selected states (Oyo and Bayelsa) in Nigeria with each state representing a different geographic and linguistic–ethnic region in the southern part of the country.

### Study population

The study was conducted in two states in Nigeria (Oyo and Bayelsa). The two states were purposively selected with each representing a different geographic and linguistic–ethnic region in the southern part of the country, Oyo in the southwest and Bayelsa in south–south with malaria prevalence of 7.6% and 3.9%, respectively [8]. Oyo state is made up of 33 LGAs out of which 12 are rural, while Bayelsa state consists of eight LGAs with only one considered urban. Two rural LGAs were selected in each state due to financial constraint. In Oyo, Ibarapa East and Kajola were randomly selected from the list of 12 rural LGAs. In Bayelsa, the LGAs were further stratified by landed and riverine, with one landed rural LGA (out of three) and one riverine LGA (out of four) selected for inclusion in the study.

### Sample size

The sample size for the study was calculated using data from a previous study on PPMV performance [16]. This study was designed to detect an 8% absolute difference or a 40% relative difference in PPMV practice with 80% power at a 5% level of significance. In order to adjust for loss of power due to clustering of PPMVs within the LGAs, a design effect of 2 was added to the sample size calculation, which assumes that the variability, intra-cluster correlation (ICC), among PPMVs within an LGA is 0.14. Applying this to the previously calculated sample size, the final sample was 30 PPMVs in each LGA. The number of PPMVs recruited per LGA was increased by 30%, for a total sample of 40 PPMVs, per LGA to account for potential refusal rate. Within each LGA, a list of PPMVs was provided by the local chapter of the national association of patent and proprietary medicine dealers (NAPPMED).

### Tools and data collection

Data collection was carried out between June and August of 2013. Data were collected from PPMV Association members on their knowledge, testing and treatment practices, based on the 2011 National Policy on Malaria Diagnosis and Treatment. A PPMV knowledge survey was used to elicit information on PPMV demographics (training/education, registration, and experience), malaria knowledge and methods of improving PPMV malaria testing and treatment practices. The question items in questionnaire except the demographics were structured using True or False and Yes or No format. The data collection tools were first developed in English, then translated into the local languages (Yoruba and Pidgin) and back translated into English. The tool was pre-tested by trained interviewers and necessary changes made prior to data collection.

### Data collection

In order to determine PPMVs knowledge of malaria diagnosis and treatment, PPMVs were asked about their knowledge of anti-malarial policies, government-recommended anti-malarial treatments and their correct dosages, and common danger signs for malaria that indicate severe malaria. In addition, PPMVs were asked how they would diagnose and treat a series of fever cases using case scenarios.

### Data management and analysis

The questionnaires were reviewed for random and systematic errors, and corrections were made. Data were cleaned and coded prior to data entry, which was done using Statistical Package for Social Sciences (SPSS) version 21.0. Descriptive statistics with mean, standard deviation, median, and range were generated; the findings were summarized and presented in tables.

### Ethical considerations

The proposal was reviewed and approved by the Research and Ethics Committees of the University of Ibadan/University College Hospital (NHREC/05/01/2008a; UI/EC/13/0032) and Niger Delta University Teaching Hospital (NDUTH/REC/2013/011) before the commencement of data collection.

## Results

A total of 160 PPMVs, 40 from each LGA, completed the survey. All of those invited to participate were available and none declined to take part in the study making a response rate of 100%.

### PPMV demographics

Table 1 depicts the PPMV characteristics in the four LGAs. A large difference in gender composition exists between PPMVs in the two states with a greater proportion of males in the two Bayelsa state LGAs (Brass 52.5%, Ogbia 62.5%), and females in Oyo state LGAs (Ibarapa East 77.5%, Kajola 82.5%). Educational levels of PPMVs were fairly similar in all four LGAs with the majority (> 75% in each LGA) reporting at least a senior secondary school education.

**Table 1:** Patent and proprietary medicine vendors demographic information

	Oyo		Bayelsa		Total
	Ibarapa East	Kajola	Brass	Ogbia	
Sex					
Male	9 (22.5)	7 (17.5)	21 (52.5)	25 (62.5)	62 (38.7)
Female	31 (77.5)	33 (82.5)	19 (47.5)	15 (37.5)	98 (61.3)
Age^a^	31.58 ± 6.8 (24–65)	36.33 ± 10.4 (22–66)	40.0 ± 12.3 (21–56)	35.5 ± 9.2 (23–67)	35.94 ± 10.3 (22–67)
Education level					
Adult education	0 (0.0)	0 (0.0)	1 (2.5)	0 (0.0)	1 (0.6)
Primary	0 (0.0)	5 (12.5)	3 (7.5)	2 (5.0)	10 (6.3)
Junior secondary	0 (0.0)	3 (7.5)	1 (2.5)	0 (0.0)	4 (2.5)
Senior secondary	35 (87.5)	19 (47.5)	23 (57.5)	25 (62.5)	102 (75.0)
Post-secondary (Gd 2)	1 (2.5)	0 (0.0)	1 (2.5)	0 (0.0)	2 (1.3)
Others	0 (0.0)	2 (5.0)	0 (0.0)	0 (0.0)	2 (1.3)
Tertiary	4 (10.0)	11 (27.5)	11 (27.5)	13 (32.5)	39 (24.4)
Senior secondary or more	40 (100.0)	30 (75.0)	35 (87.5)	38 (95.0)	143 (89.4)

^a^Mean ± SD

In addition to their basic education, most PPMVs (83%) participated in and successfully completed an apprenticeship program, although only 65.5% were reportedly issued a certificate. In only 26% of cases was the certificate observed in the shop (Table 2). Nearly all of the PPMVs in the Oyo state LGAs had completed an apprenticeship, while just over two-thirds in the Bayelsa state LGAs had. In addition, 25% of PPMVs had completed some type of health professional education programme: with other voluntary health workers (11.9%), auxiliary nurses (9.4%), and community health officers (6.3%) being the most common. Less than half of the total respondents in three of the four LGAs reportedly received training on malaria in the previous 3 years.

**Table 2:** Specialized PPMV education

Specialized PPMV education	Oyo		Bayelsa		Total
	Ibarapa East	Kajola	Brass	Ogbia	
	N (%)	N (%)	N (%)	N (%)	
Completed apprenticeship program	40 (100.0)	38 (95.0)	26 (65.0)	28 (70.0)	132 (82.5)
Issued apprenticeship certificate	40 (100.0)	36 (89.5)	14 (35.0)	15 (37.5)	105 (65.5)
Evidence of apprenticeship	21 (52.5)	11 (26.5)	3 (7.5)	7 (17.5)	42 (26.0)
Average years of apprenticeship^a^	2.63 ± 0.74 (2–5)	3.39 ± 0.72 (2–5)	4.2 ± 3.8 (1–18)	4.4 ± 2.6 (1–10)	3.5 ± 2.2 (1–18)
Completed any Health Professional Education Programme	4 (10.0)	6 (15.0)	17 (42.5)	13 (32.5)	40 (25.0)
Received training on malaria in the past 3 years	22 (55.0)	12 (30.0)	19 (47.5)	14 (47.5)	67 (41.9)

^a^Mean ± SD

### Knowledge of anti-malarial policies

Overall, PPMVs’ knowledge of the anti-malarial policy was poor, as less than 20% had heard of the 2011 National Policy on Malaria Diagnosis and Treatment, and fewer than 5% had seen or read a copy of the document while few 4 (2.5%) had read a pamphlet describing the 2011 policy.

### Knowledge of antimalarial treatments and danger signs

When asked to determine whether a list of different treatments was recommended by the government for the treatment of uncomplicated malaria, the majority of PPMVs in both states correctly identified artemether–lumefantrine (AL) (82.5%) and artesunate–amodiaquine (AA) (53.1%). Only very few, 5 (3.1%) knew artemisinin-based combinations and ACT only as correct treatment for malaria (Table 3). However, about half of the PPMVs also incorrectly identified, non-approved treatments such as artesunate monotherapy (53.1%) and chloroquine (46.9%). Even quinine, which is expected to be used in managing severe malaria as reflected in the policy, was thought to be recommended for routine treatment by 44.4% of PPMVs. Paracetamol and herbal fever mixture, a mixture of herbs soaked in water, were also thought to be government-recommended cures for malaria by 76.9% and 36.9% of PPMVs, respectively.

**Table 3:** PPMVs’ knowledge of government-recommended anti-malarials

Are the following drugs recommended by Government for curing malaria?	Oyo		Bayelsa		Total N = 160 N (%)
	Ibarapa East N = 40	Kajola N = 40	Brass N = 40	Ogbia N = 40	
	N (%)	N (%)	N(%)	N (%)	
ACT^a^					
Named at least one ACT	33 (82.5)	34 (85.0)	36 (90.0)	39 (97.5)	142 (88.8)
Artemether–lumefantrine	27 (67.5)	32 (80.0)	34 (85.0)	39 (97.5)	132 (82.5)
Artesunate–amodiaquine	25 (62.5)	8 (20.0)	25 (62.5)	27 (67.5)	85 (53.1)
Any ACT					
Named at least one ACT	33 (82.5)	34 (85.0)	36 (90.0)	39 (97.5)	142 (88.8)
Named at least one non-ACT	40 (100.0)	38 (95.0)	36 (90.0)	31 (77.5)	145 (90.6)
Know ACT and ACT only as correct treatment	0 (0.0)	1 (2.5)	1 (2.5)	3 (7.5)	5 (3.1)
Non-ACT anti-malarials					
Named at least one non-ACT	40 (100.0)	38 (95.0)	36 (90.0)	31 (77.5)	145 (90.6)
Chloroquine	28 (70.0)	24 (60.0)	12 (30.0)	11 (27.5)	75 (46.9)
Sulfadoxine–pyrimethamine (SP)	23 (57.5)	17 (42.5)	19 (47.5)	20 (50.0)	79 (49.4)
Artesunate monotherapy	17 (42.5)	19 (47.5)	27 (67.5)	22 (55.0)	85 (53.1)
Quinine	10 (25.0)	22 (55.0)	18 (45.0)	21 (52.5)	71 (44.4)
Know ACT and ACT only as correct treatment	0 (0.0)	1 (2.5)	1 (2.5)	3 (7.5)	5 (3.1)
Other treatments					
Paracetamol	38 (95.0)	35 (87.5)	26 (65.0)	24 (60.0)	123 (76.9)
Herbal fever mixture	30 (75.0)	27 (67.5)	0 (0.0)	2 (5.0)	59 (36.9)
Other	8 (20.0)	7 (17.5)	14 (35.0)	7 (17.5)	36 (22.5)

^a^Drug treatment recommended as first-line treatment by the Government of Nigeria

### Knowledge of malaria danger signs in children

As part of the survey, the PPMVs were asked to indicate “Yes” or “No” to each of the listed danger signs of severe malaria in a child under the age of two. While the majority correctly recognized persistent fever (52.5%) and severe vomiting (50.6%), less than half had knowledge of other danger signs such as inability to eat, drink or breastfeed, inability to sit or stand and difficulty breathing/fast breathing (Table 4). When asked how they would treat a child exhibiting signs of severe malaria, the majority (65.6%) correctly reported that they would refer the child to a health facility, 26.9% would give paracetamol and 23.8% would use a tepid sponge to lower the child’s fever. Thirty-seven percent of respondents reported that they would use some other treatment including giving anti-malarial drugs (artemisinin-based combinations or others), Vitamin C, a blood tonic or oral rehydration salts. Several PPMVs also reported that they would treat the child with medical treatments.

**Table 4:** PPMVs’ knowledge of danger signs of severe malaria

	Oyo		Bayelsa		Total (N = 160)
	Ibarapa East (N = 40)	Kajola (N = 40)	Brass (N = 40)	Ogbia (N = 40)	
Child danger signs					
Persistent fever	13 (32.5)	24 (60.0)	24 (60.0)	23 (57.5)	84 (52.5)
Severe vomiting	24 (60.0)	20 (50.0)	24 (60.0)	13 (32.5)	81 (50.6)
Convulsions	15 (37.5)	14 (35.0)	18 (45.0)	14 (35.0)	61 (38.1)
Inability to sit or stand	7 (17.5)	15 (37.5)	19 (47.5)	8 (20.0)	49 (30.6)
Inability to eat, drink or breastfeed	14 (35.0)	6 (15.0)	9 (22.5)	11 (27.5)	40 (25.0)
Difficulty breathing/fast breathing	7 (17.5)	5 (12.5)	3 (7.5)	2 (5.0)	17 (10.6)
Coma	1 (2.5)	6 (15.0)	0 (0.0)	0 (0.0)	7 (4.4)
Treatment of child with severe malaria					
Refer to health facility	25 (62.5)	24 (60.0)	31 (77.5)	25 (62.5)	105 (65.6)
Give paracetamol	9 (22.5)	6 (15.0)	12 (30.0)	16 (40.0)	43 (26.9)
Place tepid sponge on child	7 (17.5)	4 (10.0)	12 (30.0)	15 (37.5)	38 (23.8)
Don’t know	1 (2.5)	0 (0.0)	0 (0.0)	0 (0.0)	1 (0.6)
Others	19 (47.5)	16 (40.0)	13 (32.5)	12 (30.0)	60 (37.5)

### Malaria diagnosis and treatment case studies

As part of the PPMV knowledge assessment, PPMVs were asked how they would diagnose and treat three suspect uncomplicated malaria cases: a child, an adult man and a pregnant woman. Less than 10% of PPMVs stated that they would take a blood sample or use an RDT to diagnose malaria (Table 5). The proportion was highest for an adult male case (7.5%) and lowest for a child (1.9%). Over half of PPMVs stated they would treat adult male (65.0%) and child (58.1%) malaria cases with an artemisinin-based combination. However, when asking what dose they would provide, the proportion who reported the correct treatment and dose fell to 46.9% and 33.1% for an adult male and a child, respectively.

**Table 5:** Malaria diagnosis and treatment case studies

Case studies	Oyo		Bayelsa		Total N = 160 N (%)
	Ibarapa East N = 40	Kajola N = 40	Brass N = 40	Ogbia N = 40	
	N (%)	N (%)	N (%)	N (%)	
Treatment of child under 5					
Diagnosis method					
Blood sample/RDT	0 (0.0)	0 (0.0)	2 (5.0)	1 (2.5)	3 (1.9)
Temperature with hand	28 (70.0)	18 (45.0)	10 (25.0)	9 (22.5)	65 (40.6)
Temperature with thermometer	3 (7.5)	12 (30.0)	19 (47.5)	23 (57.5)	57 (35.6)
Refer to health facility	3 (7.5)	4 (10.0)	1 (2.5)	2 (5.0)	10 (6.3)
No diagnosis method	0 (0.0)	0 (0.0)	2 (5.0)	0 (0.0)	2 (1.3)
Others	6 (15.0)	6 (15.0)	6 (15.0)	5 (12.5)	23 (14.4)
Treatment					
Treatment with ACT	21 (52.5)	23 (57.5)	21 (52.5)	28 (70.0)	93 (58.1)
Treatment with ACT and dosage correct	9 (22.5)	8 (20.0)	17 (42.5)	19 (47.5)	53 (33.1)
Adult male					
Diagnosis method					
Blood sample/RDT	0 (0.0)	2 (5.0)	3 (7.5)	7 (17.5)	12 (7.5)
Temperature with hand	14 (35.0)	9 (22.5)	6 (15.0)	2 (5.0)	31 (19.4)
Temperature with thermometer	7 (17.5)	11 (27.5)	0 (0.0)	0 (0.0)	18 (11.3)
Refer to health facility	4 (10.0)	4 (10.0)	18 (45.0)	18 (45.0)	44 (27.5)
Others	15 (37.5)	14 (35.0)	13 (32.5)	13 (32.5)	55 (34.4)
Treatment					
Treatment with ACT	22 (55.0)	19 (47.5)	32 (80.0)	31 (77.5)	104 (65.0)
Treatment with ACT and dosage correct	12 (30.0)	10 (25.0)	27 (67.5)	26 (65.0)	75 (46.9)
Pregnant woman, 2nd trimester					
Diagnosis method					
Blood sample/RDT	2 (5.0)	0 (0.0)	0 (0.0)	3 (7.5)	5 (3.1)
Temperature with hand	7 (17.5)	2 (5.0)	2 (5.0)	2 (5.0)	13 (8.1)
Temperature with thermometer	6 (15.0)	18 (45.0)	8 (20.0)	17 (42.5)	49 (30.6)
Refer to health facility	14 (35.0)	9 (22.5)	26 (65.0)	11 (27.5)	60 (37.5)
Others	11 (27.5)	11 (27.5)	4 (10.0)	7 (17.5)	33 (20.6)
Treatment					
Treatment with ACT	9 (22.5)	22 (55.0)	3 (7.5)	7 (17.5)	41 (25.6)
Treatment with ACT and dosage correct	5 (12.5)	10 (25.0)	1 (2.5)	6 (15.0)	22 (13.8)
Prevention					
*What would you give to a pregnant woman to prevent malaria, if anything?*					
Treatment with SP	4 (10.0)	14 (35.0)	8 (20.0)	8 (20.0)	34 (21.3)
Treatment with SP and dosage correct	1 (2.5)	6 (15.0)	6 (15.0)	6 (15.0)	19 (11.9)

For pregnant women in their second trimester, PPMVs were less likely to state that they would treat them with ACT (25.6%) and use the correct dose (13.9%). Notably, knowledge of how to treat a pregnant woman for malaria falls far below knowledge for children under five and adult men. Finally, when asked what they would provide to a pregnant woman to prevent malaria, only 34 (21.3%) reported that they would give sulfadoxine–pyrimethamine (SP) and 19 (11.9%) knew to give SP at the correct dosage.

## Discussion

With 23.0% of the global deaths due to malaria, Nigeria accounts for a greater proportion of malaria-related deaths than any other country in the world [9]. Given the prevalence of PPMVs, particularly within the rural communities, and their role as a first point of care for many Nigerians, they should be included as a critical component in any national strategy to reduce the burden of malaria. This study demonstrates that while the majority of PPMV members were unaware of the 2011 National Policy on Malaria Diagnosis and Treatment, 82.5% knew that ACT was a government recommended treatment for malaria and over 58% would recommend artemisinin-based combinations for a child or adult male with malaria. However, fewer would recommend ACT at the correct dosage, with only 46.9% recommending the correct adult male dosage and 33.1% the correct child dosage.

While knowledge of ACT as the correct first-line treatment was high, many PPMVs also continued to believe that several non-ACT medicines are effective treatments for curing malaria—with 53.1% and 46.9% reporting artesunate monotherapy and chloroquine as government recommended first-line anti-malarial treatments, respectively. Similar findings were reported in studies conducted in Nasarawa and Oyo states and south Eastern Nigeria—where few febrile patients received ACT (Oladepo et al. pers. commun.). It is not enough for PPMVs to know that ACT is the government-recommended treatment, they must also be taught that other treatments are not effective.

This study also found that knowledge of signs of severe malaria in children, prevention and treatment of malaria in pregnant women were low. Just over half knew the most common signs of severe malaria, and less than a quarter would recommend SP to prevent malaria in a pregnant woman and would recommend ACT to treat malaria in a pregnant woman in her second trimester. This is a major concern, given the high levels of malaria-related complications and mortality among these vulnerable groups. In addition, knowledge of malaria diagnosis using RDTs was also low. Most often PPMVs reported that they would diagnose malaria by taking a temperature either by hand or with a thermometer. Rarely did any PPMV report that they would use an RDT for any case.

Systematic reviews have shown that private retail outlets can be effectively capacitated to conduct RDTs for malaria and to provide correct treatment for diagnosed cases [15–17]. Interventions that provided longer training, frequent supervision and addressed both supply and demand-side factors were more effective at changing provider practice. Although the available evidence suggests that well-designed interventions can improve the quality of malaria case management at PPMV shops, only 41.9% of PPMVs in this study had received training on malaria in the previous 3 years.

## Conclusions

PPMVs have low knowledge of signs of severe malaria in children and the prevention and treatment of malaria in pregnant women. PPMVs provided ACT with correct dosage advice during clients’ visits, but did not use RDTs to diagnose malaria in any of the clients. PPMVs were being eager to receive additional training on malaria treatment, and welcomed the idea of adding malaria testing using RDTs to the services they provide.

In light of the findings in this study, the authors recommend that further training and support intervention be conducted to equip PPMVs to safely and effectively use RDTs to diagnose malaria and provide appropriate treatment with artemisinin-based combinations. This would contribute to enhancing the delivery of quality malaria testing and treatment and improve health service provision among PPMVs in line with government guidelines and best malaria treatment practices. With the large numbers of PPMVs nationally, novel methods of building PPMV capacity are needed to quickly achieve scale. As many PPMVs are members of their local NAPPMED chapter, this association structure could be used as a platform for training and support (Oyeyemi et al. pers. commun.). However further research is needed to determine how best to leverage this association.

## Abbreviations

AA: artesunate–amodiaquine
ACT: arteminisin-based combination therapy
AL: arthemeter–lumefantrine
AM: artesunate monotherapy
ICC: intra-cluster correlation
ITN: insecticide-treated nets
LGA: local government area
NAPPMED: National Association of Patent and Proprietary Medicine Dealers
NMCP: National Malaria Control Programme
PPMV: patent and proprietary medicine vendors
RDT: rapid diagnostic testing
SP: sulfadoxine–pyrimethamine
